# Long non-coding RNA PAXIP1-AS1 facilitates cell invasion and angiogenesis of glioma by recruiting transcription factor ETS1 to upregulate KIF14 expression

**DOI:** 10.1186/s13046-019-1474-7

**Published:** 2019-12-10

**Authors:** Haiyang Xu, Guifang Zhao, Yu Zhang, Hong Jiang, Weiyao Wang, Donghai Zhao, Hongquan Yu, Ling Qi

**Affiliations:** 1 Department of Oncological Neurosurgery, First Hospital of Jilin University, No. 71, Xinmin Street, Changchun, 130021 Jilin Province People’s Republic of China; 2 Qingyuan People’s Hospital, The Sixth Affiliated Hospital of Guangzhou Medical University, B24 Yinquan South Road, Qingyuan, 511518 Guang dong Province People’s Republic of China; 3 Department of Pathophysiology, Jilin Medical University, No. 5, Jilin Street, Jilin, 132013 Jilin Province People’s Republic of China; 4 Department of Neurovascular, First Hospital of Jilin University, Changchun, 130021 People’s Republic of China; 5 Department of Ophthalmology, China-Japan Union Hospital of Jilin University, Changchun, 130033 People’s Republic of China

**Keywords:** Long non-coding RNA, PAXIP1-AS1, ETS1, KIF14, Glioma, Migration, Invasion, Angiogenesis

## Abstract

**Background:**

Gliomas are common life-threatening cancers, mainly due to their aggressive nature and frequent invasiveness and long non-coding RNAs (lncRNAs) are emerging as promising molecular targets. Therefore, we explored the regulatory mechanisms underlying the putative involvement of the lncRNA PAX-interacting protein 1- antisense RNA1/ETS proto-oncogene 1/kinesin family member 14 (PAXIP1-AS1/ETS1/KIF14) axis in glioma cell invasion and angiogenesis.

**Methods:**

Firstly, we identified differentially expressed lncRNA PAXIP1-AS1 as associated with glioma based on bioinformatic data. Then, validation experiments were conducted to confirm a high expression level of lncRNA PAXIP1-AS1 in glioma tissues and cells, accompanied by upregulated KIF14. We further examined the binding between lncRNA PAXIP1-AS1, KIF14 promoter activity, and transcription factor ETS1. Next, overexpression vectors and shRNAs were delivered to alter the expression of lncRNA PAXIP1-AS1, KIF14, and ETS1 to analyze their effects on glioma progression in vivo and in vitro.

**Results:**

LncRNA PAXIP1-AS1 was mainly distributed in the nucleus of glioma cells. LncRNA PAXIP1-AS1 could upregulate the KIF14 promoter activity by recruiting transcription factor ETS1. Overexpression of lncRNA PAXIP1-AS1 enhanced migration, invasion, and angiogenesis of human umbilical vein endothelial cells in glioma by recruiting the transcription factor ETS1 to upregulate the expression of KIF14, which was further confirmed by accelerated tumor growth in nude mice.

**Conclusions:**

The key findings of this study highlighted the potential of the lncRNA PAXIP1-AS1/ETS1/KIF14 axis as a therapeutic target for glioma treatment, due to its role in controlling the migration and invasion of glioma cells and its angiogenesis.

## Background

Gliomas are among the most common primary tumors of the central nervous system, exhibiting multiple heterogeneous sub-regions [1]. High-grade gliomas, such as glioblastoma, are fatal cancers with unfavorable prognoses, comprising the leading cause of brain-tumor related deaths, whereby limited improvement in treatment outcomes has been achieved despite recent advances [2, 3]. The prognoses for advanced gliomas are very poor, with less than 5.5% of cases surviving over five years, owing primarily to challenges caused by their infiltrative nature and drug resistance [4, 5]. Glioblastoma causes pathological changes in the brain vasculature, which accelerates the progression and therapeutic resistance, while the altered brain vasculature also increases the aggressiveness of the tumor [6]. Emerging research has focused on identifying molecular mechanisms that may be leveraged for diagnostic and prognostic purposes to improve clinical decisions, and hence, the long-term outcomes [7, 8].

Long non-coding RNAs (lncRNAs) have been implicated in the pathogenesis, progression, and phenotype development of gliomas, on the basis of a growing body of evidence that delineates the functional relevance of lncRNA dysregulation in glioma [9]. LncRNAs represent a heterogeneous family of functional molecules with a length over 200 nucleotides, exhibiting little or no coding potential [10]. Interestingly, multiple mechanistic studies have associated lncRNAs with significant roles in mediating key biological processes such as cell differentiation, growth, and immune responses [11, 12]. Thus, aberrantly expressed lncRNAs have the potential to influence cellular behaviors and ultimately stimulate carcinogenesis [13]. Recent studies have presented evidence demonstrating the critical roles of lncRNAs in glioma development [14, 15]. Weirick et al., demonstrated lncRNA PAXIP1-AS1 as a critical mediator of cell death, and its silencing was found to facilitate cell survival [16]. In glioma, the abnormal expression of KIF14 has been demonstrated, which is further elevated with increased aggressiveness [17]. The transcription factor ETS1, acts as an oncogene and has a key role to play in glioma development, suggesting that its inhibition may comprise a potential therapeutic strategy for glioma [18]. Thus, a hypothesis that the lncRNA PAXIP1-AS1/ETS1/KIF14 axis has functional relevance in glioma pathology was framed and investigated.

## Materials and methods

### Ethics statement

The study was conducted with the approval of the Ethics Committee of First Hospital of Jilin University. All participating patients provided written informed consent. Nude mice used for in vivo experimental animal studies were cared for in accordance with a protocol approved by the Laboratory Animal Care and Use Committee of First Hospital of Jilin University.

### Microarray data

A glioma gene-expression microarray dataset (GSE104291) and its probe annotation files were downloaded from the Gene Expression Omnibus database (https://www.ncbi.nlm.nih.gov/geo/). Differentially expressed genes were determined in the R software environment, with the threshold as |log2FoldChange| > 1 and *P.* value < 0.05.

### Tissue sample and cell culture

This study included 44 patients diagnosed with glioma who underwent primary surgery in First Hospital of Jilin University between July 2011 and April 2015. Normal brain tissue samples were obtained from 5 patients with severe head trauma who required partial removal of normal brain tissue. None of the included patients had received chemotherapy or radiation before resection. All tumors were classified according to World Health Organization criteria for central nervous system tumors and frozen immediately after surgery until analysis. Treatment for all the glioma patients included in the study was based on the guidelines of National Comprehensive Cancer Network. All patients were eligible for clinical follow-up. The patient’s overall survival was calculated from the date of primary surgery to the date of patient’s death.

Four glioma cell lines TJ905, HS 683, H4, SHG-44, and normal human astrocytes (HAs) were purchased from the National Infrastructure of Cell Line Resource (http://www.cellresource.cn/index.aspx). The cells were cultured in Dulbecco’s modification of Eagle’s medium (DMEM) (Gibco, Grand Island, NY, USA) containing 10% fetal bovine serum (FBS), 100 μg/mL streptomycin, 100 U/mL penicillin at 37 °C with 5% CO_2_ and 95% saturated humidity. The cells were passaged when cell confluence was about 80%.

### Cell transfection

Glioma cells were transfected with each of the following; negative control of overexpression vector (oe-NC) + negative control of shRNA (sh-NC), overexpression vector (oe)-PAXIP1-AS1 + sh-NC, oe-NC + sh-PAXIP1-AS1, oe-NC, oe-PAXIP1-AS1, sh-NC, sh-PAXIP1-AS1, oe-PAXIP1-AS1 + sh-ETS1, oe-PAXIP1-AS1 + sh-KIF14, oe-PAXIP1-AS1 + oe-ETS1 + sh-NC, and oe-PAXIP1-AS1 + oe-ETS1 + sh-KIF14. These plasmids were purchased from Dharmacon (Lafayette, CO, USA). The cells were plated in a 6-well plate and allowed to reach a confluence of 70–80% on the second day for transfection. The transfection was carried out using a lipofectmine 2000 kit (Invitrogen, Carlsbad, CA, USA). After 8 h of transfection, the medium was renewed and the cells were incubated for 48 h, when the cell were harvested and used for subsequent experiments.

### Quantification of gene expression

Total RNA was extracted with Trizol method (15,596,026, Invitrogen), and was reverse transcribed into cDNA using a reverse transcription kit (RR047A, Takara, Kyoto, Japan). Reverse transcription quantitative polymerase chain reaction (RT-qPCR) using a SYBR Premix EX Taq kit (RR420A, Takara) and a real-time PCR instrument (ABI7500, ABI, Foster City, CA, USA) was performed. The primers were synthesized by Sangon Biotech Co., Ltd., (Shanghai, China). The primer sequences are listed in Table 1. Glyceraldehyde-3-phosphate dehydrogenase (GAPDH) was considered as the reference housekeeping gene and the relative expression level of the amplified product was calculated by the 2^-ΔΔCt^ method [19].

**Table 1:** Primer sequences for qRT-PCR

Gene	Primer sequence
PAXIP1-AS1	Forward: 5′-GAAGTTGGGAGAAGAAAT-3’
	Reverse: 5′-AGTGTACCGCAGAGTAAT-3’
GAPDH	Forward: 5′-GCACCGTCAAGGCTGAGAAC-3’
	Reverse: 5′-TGGTGAAGACGCCAGTGGA-3’

### Fluorescence in situ hybridization (FISH)

Subcellular localization of lncRNA PAXIP1-AS1 was identified using the FISH assay performed according to the standard instructions of Ribo™ lncRNA FISH Probe Mix (Red) (C10920, RiboBio Co., Ltd., Guangzhou, Guangdong, China). The cells were seeded (6 × 10^4^ cells/well) in a 24-well culture plate and cultured until cell confluence reached 60–70%. The cells were then fixed in 4% paraformaldehyde, washed, and permeabilized. Pre-hybridization solution was employed to seal the plate. After the pre-hybridization solution was discarded, the cells were hybridized overnight at 37 °C with the probe hybridization solution containing anti-PAXIP1-AS1 nucleotide (Wuhan Genecreate Co., Ltd., Wuhan, Hubei, China) without light exposure. Following elution, the cells were stained with 4′,6-diamidino-2-phenylindole solution, rinsed, and mounted in nail polish for fluorescence microscopy (Olympus, Japan) and five different fields of view were selected for observation and photography.

### RNA immunoprecipitation (RIP)

The binding of lncRNA PAXIP1-AS1 to the transcription factor ETS1 protein was assessed using an RIP kit (Millipore Corp., Bedford, MA, USA). The cells were lysed in an ice bath, and centrifuged to collect the supernatant. The cell extract was co-precipitated by incubation with the antibody. The magnetic beads were washed and re-suspended in RIP Wash Buffer. Next, the appropriate antibody for binding was added according to each experimental group. The magnetic bead-antibody complex was washed, resuspended in RIP Wash Buffer, and incubated with the cell extract overnight at 4 °C. Thereafter, the magnetic bead-protein complex was harvested. The sample was digested with proteinase K to extract RNA for subsequent PCR detection. The antibody used for RIP was ETS1 (ab225868, 1: 200, Abcam, Cambridge, UK), and Immunoglobulin G (IgG) (ab172730, 1: 100, Abcam) was regarded as a NC.

### Western blot assay

The total protein in tissues or cells was extracted with Radio Immunoprecipitation Assay lysis buffer containing phenylmethylsulfonyl fluoride. Following incubation on ice for 30 min, the samples were centrifuged and the supernatant was collected. Sodium dodecyl sulfate-polyacrylamide gel electrophoresis was performed and the separated protein was transferred to a polyvinylidene fluoride (PVDF) membrane, which was blocked in 5% skim milk powder at room temperature for 1 h. The PVDF membrane was then incubated overnight at 4 °C with diluted primary antibodies; rabbit anti-ETS1 (ab225868, 1: 1000, Abcam), rabbit anti-KIF14 (ab71155, 1: 2000, Abcam), rabbit anti-E-Cadherin (ab40772, 1: 10000, Abcam), rabbit anti-N-Cadherin (ab18203, 1: 1000, Abcam), rabbit anti-matrix metalloproteinase-2 (MMP-2) (ab37150, 1: 1000, Abcam), rabbit anti-vascular endothelial growth factor-A (VEGF-A) (ab52917, 1: 10000, Abcam), and GAPDH (ab9485, 1: 2500, Abcam) as the internal reference. The membrane was then incubated with horseradish peroxidase (HRP)-labeled secondary anti-goat anti-rabbit IgG H&L (HRP) antibody (ab97051, 1:2000, Abcam) for 1 h. The proteins were visualized by the enhanced chemiluminescence Fluorescence Detection Kit (BB-3501, Amersham, Little Chalfont, Buckinghamshire, UK). The images were captured in a gel imager, photographed using the Bio-Rad image analysis system (BIO-RAD, USA), and analyzed by Quantity One v4.6.2 software. The relative protein level was expressed by the gray value of the corresponding protein band to that of the GAPDH protein band.

### Dual luciferase reporter gene assay

To examine the effect of lncRNA PAXIP1-AS1 on KIF14 promoter activity, oe-NC, oe-PAXIP1-AS1, sh-NC, and sh-PAXIP1-AS1 were respectively co-transfected into TJ905 cells with KIF14-2Kb luciferase reporter plasmid. Cells were harvested and lysed after transfection for 48 h. Using a luciferase assay kit (K801–200, Biovision), a luciferase reporter gene assay was performed, utilizing a dual luciferase reporter gene analysis system (Promega, Madison, WI, USA). Renilla luciferase was regarded as an internal reference gene. The activation degree of the target reporter gene was determined based on the ratio of the relative luciferase units of firefly luciferase divided with that of Renilla luciferase.

Bioinformatic analysis using UCSC (http://genome.ucsc.edu/) and JASPAR (http://jaspar.genereg.net/) web-based databases predicted that the ETS1 protein was most likely to bind to two sites of KIF14 DNA. To verify the specific site of ETS1 protein binding to KIF14 DNA, a recombinant luciferase reporter vector with the short or mutant binding site was co-transfected into TJ905 cells along with ETS1 expression vector and the dual luciferase reporter gene assay was performed as described previously.

### Chromatin immunoprecipitation (ChIP)

TJ905 cells were fixed with formaldehyde for 10 min in order to produce DNA-protein cross-linking. A sonicator was used to fragment the chromatin. After centrifugation at 12000×g for 10 min at 4 °C, the harvested cells were divided into two tubes, which were separately incubated with NC rabbit anti-IgG antibody (ab109489, 1: 300, Abcam, Shanghai China) and ETS1 antibody (Upstate Biotech., Lake Placid, NY, USA) overnight at 4 °C. The DNA-protein complex was precipitated with Protein Agarose/Sepharose, and centrifuged at 12000×g for 5 min, with the supernatant discarded. The non-specific complex was eluted and de-crosslinked overnight at 65 °C. The collected DNA fragments were extracted and purified by phenol/chloroform. The primer was designed to obtain an amplified product containing site 2 binding of ETS1 and KIF14 DNA promoter (Forward (F): 5′-CATGCTTTTCGTTTGACACCC-3′, Reverse (R): 5′-CTTTGCTTCCACCCGCCCCCC-3′). Another primer to obtain an amplified product containing the sequence distal from the promoter region of KIF14 DNA was designed as a NC for site 2 (F: 5′-AGTCCAAATAGAACACTCAA-3′, R: 5′-TAAAAAACAGCATAAAAAAG-3′). Taking the purified DNA fragment as an amplification template, the site 2 primer and distal primer (control) were each added and RT-qPCR assays were performed, in order to verify whether the site 2 of KIF14 DNA was the binding site of transcription factor ETS1.

Upon silencing lncRNA PAXIP1-AS1, the purified DNA fragment was obtained and used for ChIP assay using the methods as described above. With sh-NC as a control, the primer for site 2 was used to examine the enrichment after KIF14 promoter site 2 bound to ETS1 antibody.

### Transwell assays

The extracellular matrix (ECM) matrigel was diluted with serum-free medium to a final concentration of 1 mg/mL. ECM matrigel was used to coat the polycarbonate membrane of the 24-well Transwell upper chamber, which was incubated in 5% CO_2_ at 37 °C for 5 h to facilitate its solidification. After all excessive liquid was removed, the chamber was incubated with DMEM in 37 °C for 0.5 h in order to rehydrate the matrigel, and excessive culture medium was removed. This step was omitted for the migration assay. TJ905 cells were serum-starved for 24 h, detached and centrifuged. The pellet was resuspended in fresh FBS-free DMEM to a final concentration of 2.5 × 10^5^ cells/mL. Next, 0.2 mL suspension was added to the upper chamber with the hydrated membrane and 700 μL of pre-cooled DMEM containing 10% FBS was added to the lower chamber. The cells were cultured at 37 °C in 5% CO_2_ and saturated humidity for 24 h. The cells in the chamber and those on the membrane that failed to pass through were wiped off. The cells were fixed with methanol for 30 min and stained with 0.1% crystal violet for 20 min, followed by observation and photography under an inverted microscope. Five fields of view were randomly selected where the number of cells that had passed through the membrane was counted and averaged.

### Tube-like structure formation of human umbilical vein endothelial cells (HUVECs)

The frozen Matrigel was thawed overnight at 4 °C, added to each well of a pre-cooled 96-well plate and incubated at 37 °C for 60 min. A suspension of HUVECs was plated (2.5 × 10^4^ cells/well). Once the cells adhered to the well, the culture solution was replaced with the supernatant of transfected TJ905 cells and further incubated for 4–6 h. Appropriate fields of view were selected for observation and photography under a microscope.

### Xenograft in nude mice

A total of 70 BALA/C nude mice (4–6 weeks old, weighing 17–20 g), purchased from the Laboratory Animal Center of Jilin University (Changchun, Jilin, China), were kept in constant temperature (25–27 °C), and humidity (45–50%) conditions. These mice were randomly divided into 7 groups (10 in each group) for different treatments: oe-NC + sh-NC, oe-PAXIP1-AS1 + oe-ETS1 + sh-NC, oe-PAXIP1-AS1 + oe-ETS1 + sh-KIF14, oe-PAXIP1-AS1 + sh-NC, oe-NC + sh-PAXIP1-AS1, oe-PAXIP1-AS1 + sh-ETS1 and oe-PAXIP1-AS1 + sh-KIF14. These lentivirus vectors were transfected into cells followed by cell culture. When the cells grew to 80–90% confluence, these were detached, centrifuged, and resuspended to a density of 1 × 10^7^ cells/mL. Then, a 20 μL aliquot of cell suspension was inoculated into the thigh skin of the nude mice and the growth of the resultant tumor was observed and photographed. After 3 weeks, the nude mice were euthanized. The tumor was removed and weighed, and the growth curve of the xenograft tumor was plotted [20].

### Immunohistochemistry

Paraffin-embedded sections of tumors from mice in each group were dewaxed and hydrated. After soaking for 10 min in 3% H_2_O_2_, the sections were subjected to high-pressure antigen retrieval (Beyotime Institute of Biotechnology, Shanghai, China) for 90 s. The sections were blocked in 5% bovine serum albumin at 37 °C for 30 min, followed by incubation with CD31 rabbit monoclonal antibody (ab134168, 1: 250, Abcam) overnight at 4 °C. Next, the sections were incubated with secondary goat anti- rabbit IgG antibody (ab6721, 1: 1000, Abcam) at 37 °C for 30 min, followed by treatment with SAB working solution. The proteins were visualized by diaminobenzidine (Fuzhou Maixin Biotechnology Co., Ltd., Fuzhou, China). Counter-staining was done using hematoxylin (Sigma) and the sections were observed and photographed under an optical microscope. Five high-power fields were randomly selected from each section, and 100 cells per field were counted in order to calculate the positive expression rate [21]. The microvessel density (MVD) was determined based on CD31 positive cells. An endothelial cell cluster or a single endothelial cell stained in brown or brownish yellow color with clear margins with adjacent tumor cells, microvessels, and surrounding connective tissues was regarded as a MVD. If the structures were not connected, their branch structure was counted as a tubule. The results of immunohistochemistry were independently scored by two blinded individuals.

### Statistical analysis

SPSS (IBM SPSS Statistics, Chicago, IL, USA) was used for statistical analysis. The measurement data were expressed as mean ± standard deviation. For two-group comparisons, normally distributed data with homogeneity of variance and paired, time-dependent design were compared using the paired *t* test, while the unpaired *t* test was applied in case of unpaired two-group data. Data comparison between multiple groups was performed using one-way analysis of variance (ANOVA) with Tukey’s post hoc test. Similar comparisons between time-dependent measurements were performed with repeated measures ANOVA, followed by Bonferroni post hoc test. The survival rate of the patients was calculated using the Kaplan-Meier method, and univariate analysis was performed by Log-rank test. The difference was considered statistically significant at *p <* 0.05.

## Results

### LncRNA PAXIP1-AS1 was highly expressed in glioma and associated with poor prognosis

**Fig. 1:**
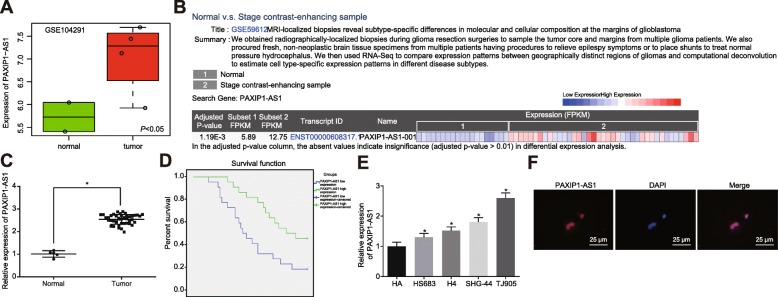
LncRNA PAXIP1-AS1 is highly expressed in glioma and is associated with poor prognosis of patients with glioma. **a**, LncRNA PAXIP1-AS1 expression in glioma-related gene-expression dataset GSE104291. **b**, The expression of lncRNA PAXIP1-AS1 in glioma within the CRN database, 1 = normal tissue, 2 = glioma tissue. **c**, The expression of lncRNA PAXIP1-AS1 within the glioma tissues (*n* = 44) and normal brain tissues (*n* = 5) was examined by RT-qPCR, * *p <* 0.05 vs. normal brain tissues. **d**, Kaplan-Meier survival analysis of the correlation of lncRNA PAXIP1-AS1 expression with the prognosis of glioma patients, and Log-rank analysis for survival difference. **e**, RT-qPCR detection of the expression of lncRNA PAXIP1-AS1 in four glioma cell lines TJ905, HS 683, H4, SHG-44, and normal HAs, * *p <* 0.05 vs. HAs. **f**, RNA-FISH assay for the subcellular localization of lncRNA PAXIP1-AS1 in TJ905 cells (400 ×). The values are measurement data and expressed as mean ± standard deviation. Differences between two groups were compared using paired *t* test, and those between multiple groups were compared using one-way ANOVA with Tukey’s post hoc test. The Kaplan-Meier method was used to calculate the patient survival rate, and log-rank test was done for univariate analysis. Cell experiments were repeated three times independently

A high expression level of lncRNA PAXIP1-AS1 was found in glioma tissues by differential expression analysis of the gene-expression dataset GSE104291 (Fig. 1a), together with CRN database analysis (http://syslab4.nchu.edu.tw/) (Fig. 1b). To verify these results from the bioinformatic prediction, the expression of lncRNA PAXIP1-AS1 in glioma tissues (*n* = 44) and normal brain tissues (*n* = 5) was measured by RT-qPCR (Fig. 1c). Relative to normal brain tissues, the expression of lncRNA PAXIP1-AS1 was significantly upregulated in glioma tissues (*p <* 0.05). Kaplan-Meier analysis of the association between lncRNA PAXIP1-AS1 expression and survival of glioma patients (Fig. 1d) revealed that the overall survival of glioma patients with high lncRNA PAXIP1-AS1 expression was lower than that in patients with low lncRNA PAXIP1-AS1 expression (*p <* 0.05). Next, RT-qPCR examined the expression of lncRNA PAXIP1-AS1 in four glioma cell lines TJ905, HS 683, H4, SHG-44 and in normal HAs (Fig. 1e). As compared to HA, the four human glioma cell lines displayed higher expression levels of lncRNA PAXIP1-AS1 (*p <* 0.05), and the most significant expression upregulation was identified in TJ905 cells. Therefore, TJ905 cells were selected for subsequent cell experiments. The subcellular localization of lncRNA PAXIP1-AS1 in TJ905 cells was identified as within the nucleus, when examined by RNA-FISH (Fig. 1f). Taken together, these findings demonstrated an aberrant expression of lncRNA PAXIP1-AS1 was evident in glioma cells and tissues and was also significantly associated with survival outcomes of patients with glioma.

### Silencing of lncRNA PAXIP1-AS1 inhibited migration, invasion and angiogenesis of glioma cells

**Fig. 2:**
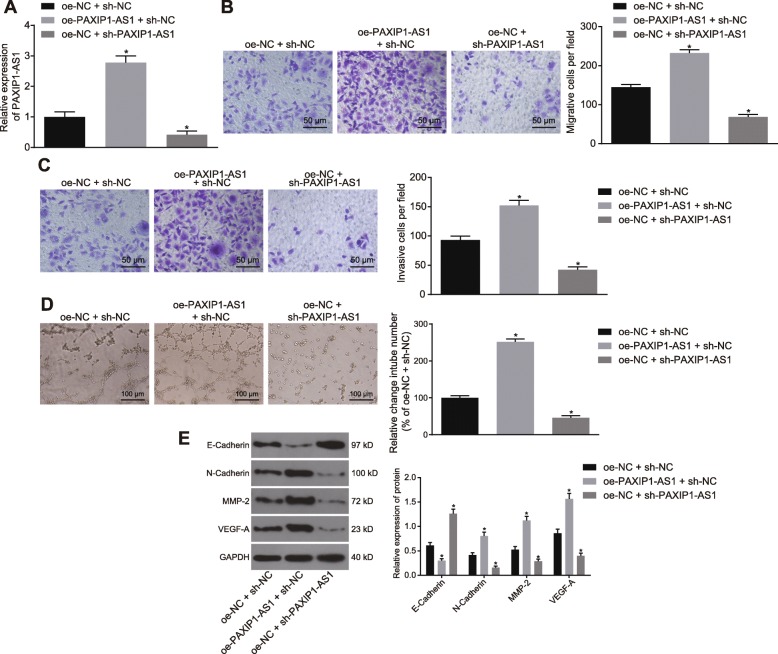
Silencing of lncRNA PAXIP1-AS1 inhibits migration, invasion, and angiogenesis of glioma cells. **a**, RT-qPCR was used to identify the efficiency of overexpressing or silencing lncRNA PAXIP1-AS1 in TJ905 cells. **b**, Transwell assay to detect the migration ability of TJ905 cells (200 ×). **c**, Transwell assay to detect the invasive ability of TJ905 cells (200 ×). **d**, The tube formation assay to detect angiogenesis in HUVECs cocultured with TJ905 cells (100 ×). **e**, Western blot analysis to determine the expression of E-Cadherin, N-Cadherin, MMP-2 and VEGF-A normalized to GAPDH in TJ905 cells. * *p <* 0.05 vs. the oe-NC + sh-NC group. The values are measurement data and expressed as mean ± standard deviation. Differences among multiple groups were compared using one-way ANOVA with Tukey’s post hoc test. Cell experiments were repeated three times independently

The effects of lncRNA PAXIP1-AS1 on the biological characteristics of glioma cells were examined by inducing its overexpression or silencing. The efficiency of overexpression or silencing lncRNA PAXIP1-AS1 in TJ905 cells met the requirements for further experiments (*p <* 0.05) (Fig. 2a). The Transwell assay (Fig. 2b, c) indicated that overexpression of lncRNA PAXIP1-AS1 promoted the migration and invasion of TJ905 cells, while silencing of lncRNA PAXIP1-AS1 inhibited their migration and invasion (*p <* 0.05). The tube formation assay using HUVECs (Fig. 2d) demonstrated that overexpression of lncRNA PAXIP1-AS1 promoted angiogenesis, and silencing reversed this situation (*p <* 0.05). The expression of epithelial marker (E-Cadherin), as well as interstitial markers (N-Cadherin, MMP-2 and VEGF-A) was examined by Western blot analysis (Fig. 2e), which indicated that the treatment of oe-PAXIP1-AS1 + sh-NC resulted in decreased expression of E-Cadherin and increased expression of N-Cadherin, MMP-2, and VEGF-A, as compared with the treatment of oe-NC + sh-NC. Besides, treatment with oe-NC + sh-PAXIP1-AS1, when compared with the treatment with oe-NC + sh-NC, led to an elevated expression of E-Cadherin, reduced expression of N-Cadherin, MMP-2 and VEGF-A (*p <* 0.05). Together, these data indicated that silencing lncRNA PAXIP1-AS1 exerted inhibitory effects on glioma cell migration, invasion, and angiogenesis.

### LncRNA PAXIP1-AS1 upregulated the expression of KIF14 gene by recruiting the transcription factor ETS1

**Fig. 3:**
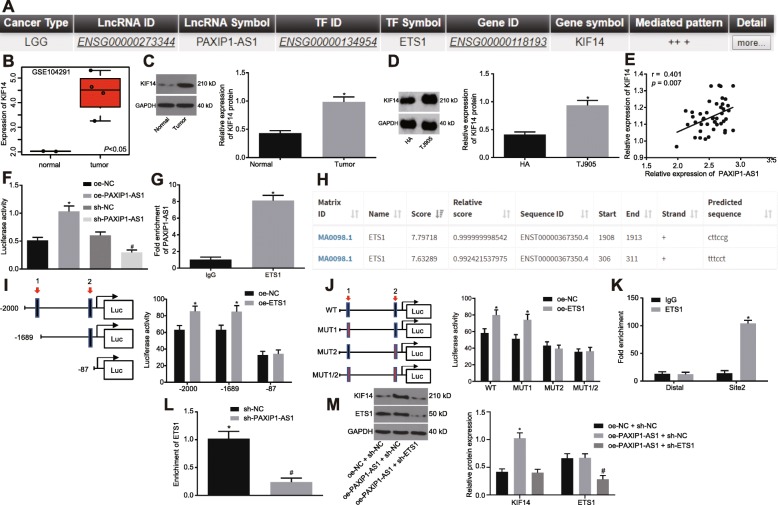
LncRNA PAXIP1-AS1 regulates the expression of KIF14 by recruiting the transcription factor ETS1. **a**, Results of bioinformatics predictions. **b**, KIF14 expression in the glioma-related microarray dataset GSE104291. **c**, Western blot analysis of KIF14 expression normalized to GAPDH in glioma tissues, * *p <* 0.05 vs. the normal brain tissues. D, Western blot analysis of KIF14 expression normalized to GAPDH in TJ905 cells, * *p <* 0.05 vs. HAs. E, Pearson’s correlation analysis between lncRNA PAXIP1-AS1 and KIF14 expression in glioma tissues (n = 44). **f**, The dual luciferase reporter gene assay to examine the effect of lncRNA PAXIP1-AS1 on the KIF14 promoter activity, * *p <* 0.05 vs. the oe-NC group, # *p <* 0.05 vs. the sh-NC group. **g**, RIP assay verified that lncRNA PAXIP1-AS1 could bind to transcription factor ETS1, * *p <* 0.05 vs. IgG. **h**, The two sites of the KIF14 DNA promoter region that transcription factor ETS1 most likely to bind to. **i**, The truncated KIF14 recombinant luciferase reporter vector and ETS1 expression vector were constructed for transfection into TJ905 cells for dual luciferase reporter gene assay, * *p <* 0.05 vs. the oe-NC group. **j**, The mutant KIF14 recombinant luciferase reporter vector and ETS1 expression vector were co-transfected into TJ905 cells for dual luciferase reporter gene assay, * *p <* 0.05 vs. the oe-NC group. **k**, ChIP assay for the binding ability of ETS1 at binding site 2 of KIF14 promoter region, * *p <* 0.05 vs. IgG. **l**, ChIP assay for the enrichment of KIF14 by ETS1 after silencing lncRNA PAXIP1-AS1 in TJ905 cells, # *p <* 0.05 vs. the sh-NC group. **m**, Western blot analysis for the expression of KIF14 and ETS1 normalized to GAPDH in each group, * *p <* 0.05 vs. the oe-NC + sh-NC group, # *p <* 0.05 vs. the PAXIP1-AS1 + sh-NC group. The values are measurement data and expressed as mean ± standard deviation. Comparison of normally distributed data between two paired groups with homogeneity of variance was performed using a paired *t* test. The unpaired *t* test was used to compare two sets of data from independent groups with normal distribution and homogeneity of variance. Data comparisons between multiple groups were performed using one-way ANOVA with Tukey’s post hoc test. Experiments were repeated three times independently

Bioinformatic analysis using LncMAP (http://bio-bigdata.hrbmu.edu.cn/LncMAP/), showed that lncRNA PAXIP1-AS1 could regulate the expression of KIF14 through the transcription factor ETS1 (Fig. 3a). Besides, we found that KIF14 was highly expressed in the glioma gene-expression microarray dataset GSE104291 (Fig. 3b). To further understand their effects in glioma, we examined the expression of KIF14 in glioma tissues and cells by Western blot analysis (Fig. 3c, d). The results showed that KIF14 was highly expressed in glioma tissues and cells as compared with the corresponding controls (*p <* 0.05). Furthermore, the results showed that in glioma tissues the expression of lncRNA PAXIP1-AS1 and that of KIF14 were positively correlated (*p <* 0.05) (Fig. 3e).

The dual luciferase reporter gene assay further validated the effect of lncRNA PAXIP1-AS1 on KIF14 promoter activity. The results showed that the cells treated with oe-PAXIP1-AS1 displayed higher KIF14 promoter activity than the oe-NC treatment (*p <* 0.05). The activity of KIF14 promoter in response to sh-PAXIP1-AS1 treatment was lower than that in response to sh-NC treatment (*p <* 0.05), indicating that lncRNA PAXIP1-AS1 could positively regulate the expression of the KIF14 gene (Fig. 3f). To further delineate the mechanism by which lncRNA PAXIP1-AS1 positively regulated KIF14 gene expression, RIP assay was used to examine whether lncRNA PAXIP1-AS1 could bind to transcription factor ETS1 (Fig. 3g). The lncRNA PAXIP1-AS1 binding to ETS1 was increased, as compared with IgG (*p <* 0.05), indicating that ETS1 protein could specifically bind to lncRNA PAXIP1-AS1. In order to identify the specific site of ETS1 protein binding to KIF14 DNA, UCSC (http://genome.ucsc.edu/) and JASPAR (http://jaspar.genereg.net/) were leveraged and the ETS1 protein was found most likely to bind to two sites in the KIF14 promoter region (Fig. 3h). Subsequently, the luciferase reporter gene assay (Fig. 3i, j) showed that site 2 was the specific site of ETS1 protein binding to the KIF14 promoter region. Next, the binding ability of ETS1 to the binding site 2 of the KIF14 promoter region was examined by ChIP assay in TJ905 cells (Fig. 3k). The amount of amplification products obtained when using the site 2 primers was greater than that obtained using distal primers in ETS1 protein, as compared with that in IgG (*p <* 0.05), but no significant difference was detected in the amount of amplification products between the two pairs of primers in IgG (*p* > 0.05), indicating that the site 2 of the KIF14 promoter region (cttccg) was indeed the binding site of transcription factor ETS1. To assess the role of lncRNA PAXIP1-AS1 in the regulation of transcription factor ETS1 and KIF14 DNA, we silenced lncRNA PAXIP1-AS1 in TJ905 cells used for ChIP assay (Fig. 3l). As compared to treatment of sh-NC, in the ETS1 antibody-enriched samples with the sh-PAXIP1-AS1 treatment, the amplification products obtained by RT-qPCR using KIF14 site 2 primers were diminished (*p <* 0.05).

Next, in order to further validate that lncRNA PAXIP1-AS1 regulated the expression of KIF14 by binding to the transcription factor ETS1, treatment with oe-NC + sh-NC, oe-PAXIP1-AS1 + sh-NC, and oe-PAXIP1-AS1 + sh-ETS1 was performed. The protein level of KIF14 was higher in response to oe-PAXIP1-AS1 + sh-NC than that in response to oe-NC + sh-NC (*p <* 0.05), while no significant difference was evident in ETS1 level, and levels of KIF14 and ETS1 decreased in the oe-PAXIP1-AS1 + sh-ETS1 treatment versus the treatment of oe-PAXIP1-AS1 + sh-NC (*p <* 0.05) (Fig. 3m), indicating that lncRNA PAXIP1-AS1 mediated the expression of KIF14 by recruiting the transcription factor ETS1.

### Highly expressed lncRNA PAXIP1-AS1 promoted glioma cell migration, invasion and angiogenesis by recruiting transcription factor ETS1 to upregulate KIF14 expression

In order to explore the effect of lncRNA PAXIP1-AS1 on the biological characteristics of glioma cells, oe-NC + sh-NC, oe-PAXIP1-AS1 + sh-NC, oe-PAXIP1-AS1 + sh-ETS1 and oe-PAXIP1-AS1 + sh-KIF14 were delivered to TJ905 cells and effects on migration, invasion and angiogenesis of glioma cells were examined.

**Fig. 4:**
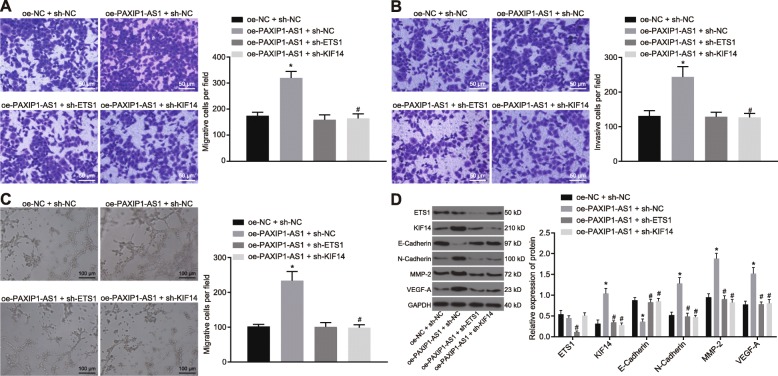
Overexpressed lncRNA PAXIP1-AS1 affects glioma cell migration, invasion, and angiogenesis by recruiting transcription factor ETS1 to upregulate KIF14 expression. **a**, Transwell assay to examine the migration ability of glioma cells (200 ×). **b**, Transwell assay to examine the invasive ability of glioma cells (200 ×). **c**, The tube formation assay to examine angiogenesis of HUVECs cocultured with TJ905 cells (100 ×). **d**, Western blot analysis for expression of E-Cadherin, N-Cadherin, MMP-2, VEGF-A, ETS1 and KIF14 normalized to GAPDH in glioma cells. * *p <* 0.05 vs. the oe-NC + sh-NC group, # *p <* 0.05 vs. the oe-PAXIP1-AS1 + oe-ETS1 + sh-NC group. The values are measurement data and expressed as mean ± standard deviation. Comparisons among multiple groups were made by one-way ANOVA with Tukey’s post hoc test. Experiments were repeated three times independently

A transwell assay was used to examine the migration and invasion ability of glioma cells (Fig. 4a, b). The migration and invasion ability of glioma cells was enhanced in response to oe-PAXIP1-AS1. The treatment of oe-PAXIP1-AS1 + sh-ETS1 or sh-KIF14 attenuated the migration and invasion ability of glioma cells (*p <* 0.05). The tube formation assay in HUVECs (Fig. 4c) suggested enhanced angiogenesis in response to the treatment of oe-PAXIP1-AS1; besides, angiogenesis was diminished in response to the treatment with oe-PAXIP1-AS1 + sh-ETS1 or sh-KIF14 (*p <* 0.05). Western blot assay examined the expression of E-Cadherin, N-Cadherin, MMP-2, VEGF-A, ETS1 and KIF14 (Fig. 4d), and demonstrated that the treatment of oe-PAXIP1-AS1 led to decreased expression of E-Cadherin and enhanced expression of N-Cadherin, MMP-2, VEGF-A and KIF14. The addition of sh-ETS1 or sh-KIF14 could reverse the effects of oe-PAXIP1-AS1 on the expression of E-Cadherin, N-Cadherin, MMP-2, VEGF-A and KIF14 (*p <* 0.05). In addition, silencing ETS1 resulted in significantly reduced ETS1 level while silencing KIF14 resulted in no significant difference on ETS1 expression. When lncRNA PAXIP1-AS1 and ETS1 were both overexpressed, malignant cell migration, invasion, and angiogenesis were promoted while the addition of silenced KIF14 could counteract the promoting effect of overexpressed lncRNA PAXIP1-AS1 and ETS1 (Additional file 1: Figure S1). Thus, overexpressed lncRNA PAXIP1-AS1 enhanced glioma cell migration, invasion, and angiogenesis by recruiting transcription factor ETS1 to upregulate KIF14 expression.

### LncRNA PAXIP1-AS1/ETS1/KIF14 axis affected the development of glioma in vivo

**Fig. 5:**
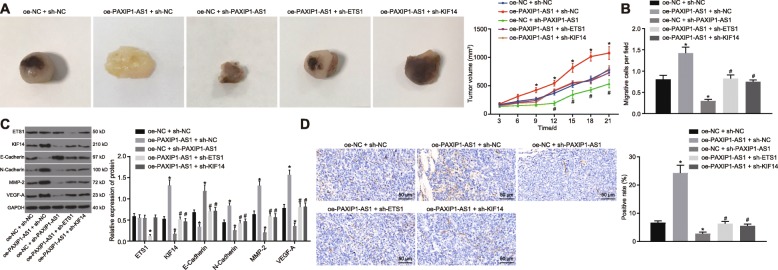
LncRNA PAXIP1-AS1/ETS1/KIF14 axis affects the development of glioma in vivo. **a**, The representative images and volume quantitation of xenograft tumors. **b**, The weight quantitation of xenograft tumor. **c**, Western blot analysis of expression of E-Cadherin, N-Cadherin, MMP-2, VEGF-A, ETS1 and KIF14 normalized to GAPDH in xenograft tumors. **d**, The expression of CD31 examined by immunohistochemistry to calculate MVD in xenograft tumors (200 ×). * *p <* 0.05 vs. the oe-NC + sh-NC group, # *p <* 0.05 vs. the oe-PAXIP1-AS1 + oe-ETS1 + sh-NC group. The values are measurement data and expressed as mean ± standard deviation. Comparisons among multiple groups were made using one-way ANOVA with Tukey’s post hoc test. Comparisons between time-based measurements were performed with repeated measures ANOVA, followed by Bonferroni post hoc test. *N* = 10

In order to further delineate the effects of the interaction between lncRNA PAXIP1-AS1, ETS1 and KIF14 on the biological characteristics of glioma cells in vivo, oe-NC + sh-NC, oe-PAXIP1-AS1 + sh-NC, oe-NC + sh-PAXIP1-AS1, oe-PAXIP1-AS1 + sh-ETS1 and oe-PAXIP1-AS1 + sh-KIF14 were each delivered into TJ905 cells and injected into nude mice in order to examine effects on tumorigenicity. The treatment with oe-PAXIP1-AS1 resulted in increased volume and weight of the tumors, as compared with the tumors treated with oe-NC + sh-NC (*p <* 0.05) (Fig. 5a, b). The volume and weight of the tumors in response to the treatment of sh-PAXIP1-AS1 were reduced, while co-treatment of oe-PAXIP1-AS1 + sh-ETS1 or KIF14 could reverse the effects of oe-PAXIP1-AS1 treatment alone (*p <* 0.05) (Fig. 5a, b). Western blot analysis examined the expression of ETS1, KIF14, E-Cadherin, N-Cadherin, MMP-2 and VEGF-A in the removed tumors (Fig. 5c). The treatment of oe-PAXIP1-AS1 led to diminished expression of E-Cadherin, accompanied by enhanced expression of KIF14, N-Cadherin, MMP-2 and VEGF-A (*p <* 0.05) while ETS1 expression was not significantly changed (*p >* 0.05). When lncRNA PAXIP1-AS1 was knocked down, the opposite results were observed (*p <* 0.05). In response to the treatment with oe-PAXIP1-AS1 + sh-ETS1 or sh-KIF14, the expression of E-Cadherin was enhanced and the expression of ETS1, KIF14, N-Cadherin, MMP-2 and VEGF-A was repressed in tumors (*p <* 0.05). Finally, we measured the expression of CD31 by immunohistochemistry to calculate MVD in the xenograft tumors (Fig. 5d). In the xenograft tumors treated with oe-PAXIP1-AS1, MVD was higher, and was lowered in the presence of sh-PAXIP1-AS1 (*p <* 0.05). Besides, the MVD in the tumors treated with oe-PAXIP1-AS1 + sh-ETS1 or sh-KIF14 was higher (*p <* 0.05). Thus, the co-overexpression of lncRNA PAXIP1-AS1 and ETS1 could accelerate tumor development and silencing KIF14 suppressed tumor growth and angiogenesis (Additional file 2: Fig. S2). These results confirmed that the interactions between lncRNA PAXIP1-AS1, ETS1 and KIF14 participated in the growth and angiogenesis of xenograft glioma tumors.

## Discussion

**Fig. 6:**
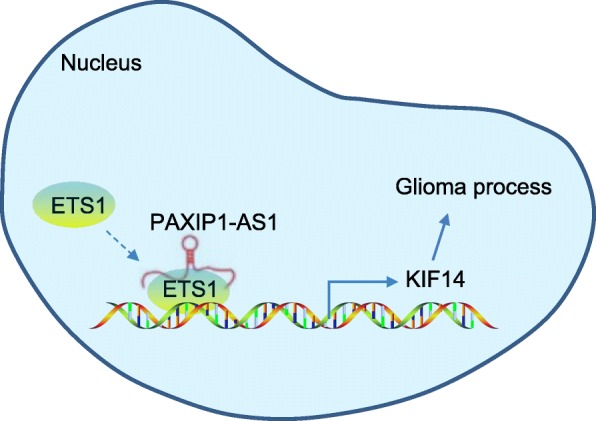
A schematic representation of the functional relevance of lncRNA PAXIP1-AS1 in glioma. LncRNA PAXIP1-AS1 upregulates the expression of KIF14 by recruiting the transcription factor ETS1 to promote the development of glioma, as reflected by enhanced glioma cell migration, invasion and angiogenesis

Glioma is typically characterized by aggressive angiogenesis and high proliferative potential, leading to unfavorable prognosis, frequent recurrence, and pronounced invasiveness [9]. An understanding of specific molecular events underpinning tumorigenesis in glioma could allow for earlier detection and improved outcomes. Recent insights into glioma tumorigenesis and development point to the relevance of lncRNAs and their functional roles [22–24]; therefore, investigating lncRNAs as prospective novel glioma biomarkers and therapeutic targets is of significance. Herein, we examined the expression and functional relevance of lncRNA PAXIP1-AS1 in glioma, which was involved with the ETS1/KIF14 axis (Fig. 6).

lncRNAs impact malignancy, including glioma, by influencing critical events such as cell proliferation, metastasis, and angiogenesis [14, 25]. Here, we revealed that the lncRNA PAXIP1-AS1 was highly expressed in glioma and also associated with poor prognosis. Aberrant lncRNA expression patterns have been associated with the histological grading of malignancy of glioma specimens and demonstrated as prognostic indicators [26, 27]. lncRNA PAXIP1 has been noted to correlate with breast cancer staging and survival, suggesting its prognostic significance in cancer [28]. While previous research concerning the mechanistic roles of lncRNA PAXIP1-AS1 in glioma is lacking, its effects on cellular and biological processes have been documented, which provides prior support for our findings. Notably, lncRNA PAXIP1-AS1 is reported to be a significant mediator of cell death [16]. Based on a series of in vitro and in vivo assays, the present study revealed that silencing of lncRNA PAXIP1-AS1 diminished the migration, invasion, and angiogenesis of glioma cells, corresponding to repressed tumor growth in nude mice. Past data suggests lncRNA PAXIP1-AS1 can have multiple downstream effects. A previous transcriptomic analysis, accompanied by functional assays, has provided evidence demonstrating a regulatory role for lncRNA PAXIP1-AS1 in idiopathic pulmonary arterial hypertension by controlling the proliferative and migratory potential of smooth muscle cells by modulating the downstream target paxillin [29]. Another prior study has highlighted the role of lncRNA PAXIP1 in chorion and amnion development, and validated its effects on DNA damage repair and transcription [30].

Here, we have demonstrated that lncRNA PAXIP1-AS1 promoted glioma cell migration, invasion and angiogenesis by recruiting transcription factor ETS1 to upregulate KIF14 expression. The transcription factor ETS1 is found to bear an essential role in the expansion of and cytokine production by group 2 innate lymphoid cells [31]. The depletion of transcription factor can result in a simulated process of secretion of autoantibodies, with the progression of autoimmune diseases [32]. It has been well established that ETS1, as a proto-oncoprotein, promotes invasive behaviors in endothelial cells and epithelial cancer cells, which is highly suggestive of unfavorable prognosis [33]. Data concerning the tumor promotive role of ETS1 in a variety of tumors has been accruing, such as in gastric cancer [34], breast cancer [35] and glioma [36]. ETS1 has also been reported to potentially function as an angiogenic mediator in ovarian cancer [37] and gastric cancer [38], which supports a role of ETS1 in controlling the angiogenesis in glioma. In glioma, high KIF14 expression in tissues has been associated with higher pathological grade, and upregulated KIF14 has been observed to predict lower overall survival [17]. In general, the kinesin family proteins are considered as potential targets for cancer therapies, owing to their roles in tumorigenesis and progression [39]. Cell behaviors in glioma could be orchestrated by lncRNA PAXIP1-AS1, due to its mechanistic function in mediating KIF14 via ETS1. Specifically, this study provides evidence suggesting that lncRNA PAXIP1-AS1 upregulates the expression of KIF14 by recruiting the transcription factor ETS1, which accelerates the development of glioma as reflected by enhanced glioma cell migration, invasion, and angiogenesis.

## Conclusion

The evidence provided by this study supports the stance that the lncRNA PAXIP1-AS1/ETS1/KIF14 axis is implicated in the aggression potential of glioma and may be targeted for the development of therapies. We note that overexpression of lncRNA PAXIP1-AS1 advances the development of glioma by recruiting the transcription factor ETS1 to increase KIF14 expression. Further studies investigating the precise mechanisms involved are warranted on the basis of these findings. Moreover, larger regulatory molecular networks must be examined in this context.

## Supplementary information

**Additional file 1: Figure S1.** LncRNA PAXIP1-AS1 affects glioma cell migration, invasion, and angiogenesis by regulating KIF14 expression. A, Transwell assay to examine the migration ability of glioma cells (200 ×). B, Transwell assay to examine the invasive ability of glioma cells (200 ×). C, The tube formation assay to examine angiogenesis of HUVECs (100 ×). D, Western blot analysis for expression of E-Cadherin, N-Cadherin, MMP-2, VEGF-A, ETS1 and KIF14 normalized to GAPDH in glioma cells. * *p <* 0.05 vs. the oe-NC + sh-NC group, # *p <* 0.05 vs. the oe-PAXIP1-AS1 + oe-ETS1 + sh-NC group. The values are measurement data and expressed as mean ± standard deviation. Comparisons among multiple groups were made by one-way ANOVA with Tukey’s post hoc test. Cell experiment was repeated three times independently.

**Additional file 2: Figure S2.** LncRNA PAXIP1-AS1/ETS1/KIF14 axis affects the development of glioma in vivo. A, Representative images and volume quantitation of xenograft tumors. B, The weight quantitation of xenograft tumors. C, Western blot analysis of expression of E-Cadherin, N-Cadherin, MMP-2, VEGF-A, ETS1 and KIF14 normalized to GAPDH in xenograft tumors. D, The expression of CD31 examined by immunohistochemistry to calculate MVD in xenograft tumors (200 ×). * *p <* 0.05 vs. the oe-NC + sh-NC group, # *p <* 0.05 vs. the oe-PAXIP1-AS1 + oe-ETS1 + sh-NC group. The values are measurement data and expressed as mean ± standard deviation. Differences among multiple groups were compared by one-way ANOVA with Tukey’s post hoc test. Comparisons between time-based measurements were performed with repeated measures ANOVA, followed by Bonferroni post hoc test. *N* = 10.

## Data Availability

The datasets generated and/or analysed during the current study are available from the corresponding author on reasonable request.

## Abbreviations

ECM: Extracellular matrix
ETS1: ETS proto-oncogene 1
FBS: Fetal bovine serum
GEO: Gene Expression Omnibus
HAs: Human astrocytes
HUVECs: Human umbilical vein endothelial cells
HUVECs: Human umbilical vein endothelial cells
KIF14: Kinesin family member 14
lncRNAs: Long non-coding RNAs
NCCN: National Comprehensive Cancer Network
oe-NC: Negative control of overexpression vector
PAXIP1-AS1: PAX interacting protein 1 antisense RNA 1
RLU: Relative luciferase unit
sh-NC: Negative control of shRNA
